# Cold Affusion in Delirium Tremens

**Published:** 1905-07-08

**Authors:** 


### COLD AFFUSION IN DELIRIUM TREMENS.

Sir Wm. Broadbent [1] draws attention to the value of cold affusion in delirium tremens. The *modus operandi* is as follows:—The patient is stripped and placed on a blanket over a waterproof sheet. Then a large bath-sponge dripping with ice-cold water is dashed violently and repeatedly on the face, neck, chest, and body. After rubbing the patient dry with a rough towel the process is repeated a second and a third time. The patient is now placed on his face and the sponge dashed on the back of the head and down the whole length of the spine two or three times, vigorous friction with a bath-towel being practised between the cold-water "attacks." The result is prompt sleep, from which the patient wakes comfortable and refreshed.

[1] Brit. Med. Jour., July 1, 1905.

